# Aging-associated transcriptional programming of mitochondrial respiration in alveolar type II epithelial cells

**DOI:** 10.3389/fcell.2026.1740661

**Published:** 2026-06-11

**Authors:** Lu Chen, Silu Hu, Xiaoju Tang, Yujun Wang, Ying Zou, Fengming Luo, Huajing Wan

**Affiliations:** 1 Department of Respiratory and Critical Care Medicine, State Key Laboratory of Respiratory Health and Multimorbidity, West China Hospital, Sichuan University, Chengdu, Sichuan, China; 2 Department of Respiratory and Critical Care Medicine, West China Hospital, Sichuan University, Chengdu, Sichuan, China; 3 Department of Respiratory and Critical Care Medicine, Frontiers Science Center for Disease-related Molecular Network, West China Hospital, Sichuan University, Chengdu, Sichuan, China

**Keywords:** aging, alveolar type II epithelial cells, bioinformatic analyses, mitochondria, transcriptional programming

## Abstract

Alveolar type II epithelial cells (AT2), notable for their high mitochondria content, are essential for maintaining pulmonary homeostasis. However, how mitochondrial function was transcriptionally programmed during AT2 aging remains poorly understood. Here, we used single-cell transcriptome data from 28 healthy human lungs spanning various ages to systematically profile age-related transcriptional changes. Our data suggest that most classical aging markers remained stable with no significant changes during AT2 aging. Using high-dimensional weighted gene co-expression network analysis (hdWGCNA), we identified a gene expression module (M1) that declines during AT2 aging and is enriched for genes involved in mitochondrial respiration. Moreover, by integrating bioinformatic and experimental approaches, we found three transcription factors (TCF7L2, FOXJ3, and TCF7) that play key regulatory roles in controlling expression of mitochondrial respiration-related genes during AT2 aging. Collectively, our findings provide both a transcriptional framework and candidate mitochondria regulators to facilitate future investigation of the causal links between AT2 mitochondrial respiration dysfunction and age-related decline in lung function.

## 1 Introduction

Alveolar type II epithelial cells (AT2) are among the most mitochondria-rich cells in the lung and serve as resident progenitors of the alveolar epithelium, capable of self-renewal and differentiation into alveolar type I (AT1) cells to maintain alveolar structure and homeostasis (Massaro et al., 1975; Piantadosi and Suliman, 2017). Given their metabolic activity, mitochondrial function is particularly critical for sustaining the diverse biological functions of AT2, including surfactant metabolism, alveolar fluid balance, innate host defense, and alveolar repair and regeneration (Hirai and Ogawa, 1986; Barkauskas et al., 2013; Kiefmann et al., 2019). Notably, the accumulation of enlarged fused mitochondria and dysregulated mitophagy were identified to be hallmarks of AT2 aging, associating with impaired regenerative capacity and surfactant homeostasis (Bueno et al., 2015; Mora et al., 2017). Moreover, such structural and functional abnormalities of mitochondria in AT2 have also been identified as a unifying pathological feature across multiple parenchymal lung diseases, including chronic obstructive pulmonary disease (COPD) and idiopathic pulmonary fibrosis (IPF) (Tuder and Petrache, 2012; Zhu et al., 2015). Collectively, these observations highlight the indispensable role of mitochondria in AT2 in maintaining alveolar homeostasis. However, our current understanding of how AT2 mitochondria are precisely regulated remains limited. In particular, identifying key molecular regulators that govern AT2 mitochondrial function will advance our knowledge of lung aging and aging-related pulmonary diseases.

Mitochondria are far more than the “powerhouses” of the cell. Accumulating evidence demonstrated that mitochondria are dynamic organelles at the crossroads of energy production, metabolism, and signal transduction, influencing nearly every aspect of cell biology and tissue homeostasis, including the production of ATP, synthesis of key metabolites, maintenance of cellular calcium homeostasis, iron metabolism, redox balance and regulation of pathogen defense, cell death, and inflammation (Duchen, 2004; Bock and Tait, 2020; Delgado and Pernas, 2025). Proteomic research demonstrated that mitochondria comprise approximately 1000–1500 different proteins encoded by both nuclear and mitochondrial genomes (Morgenstern et al., 2021). Strikingly, 99% of mitochondrial proteins are nuclear-encoded, reflecting the deep evolutionary integration between mitochondria and the host genome (Pfanner et al., 2019). Previous studies identified numerous nuclear transcription factors that regulated mitochondrial biogenesis/function in various tissues or cell types. However, only a limited number has been functionally validated in AT2, such as P53, ATF3, and KLF13 (Panduri et al., 2006; Bueno et al., 2018; Xi et al., 2025). How nuclear-encoded mitochondrial proteins are transcriptionally programmed in AT2 during different stages of adult aging remains largely unclear.

Mitochondrial dysfunction is a hallmark of aging. Deciphering how mitochondrial proteins are transcriptionally programmed in AT2 across different stages of adult aging offers key insights into mechanisms of lung degeneration and therapeutic opportunities (Cloonan et al., 2020). Recent advances in high-throughput single-cell sequencing, together with the accumulation of mitochondrial proteome resources—MitoCarta 3.0 (Rath et al., 2021), senescence database—CSGene (Zhao et al., 2016), and single-cell transcriptomic dataset of human lungs across age groups (Adams et al., 2020), provide us great opportunities to investigate mitochondrial gene expression in AT2 cells across different stages of adult aging. In parallel, the maturation of bioinformatics approaches, including high-dimensional transcriptome network analysis such as high-dimensional weighted gene co-expression network analysis (hdWGCNA) (Morabito et al., 2023), motif enrichment-based transcription factor prediction (Bailey et al., 2009; Basu et al., 2018), now enables systematic characterization of age-associated alterations in AT2 mitochondrial protein expression and the identification of nuclear transcription factors that program mitochondrial function. In this study, by combining bioinformatic prediction with cellular experiments, we characterized the age-related expression patterns of AT2 and found that the expression of genes involved in mitochondrial respiration decreased significantly in AT2 with aging. In addition, we identified the transcription factors FOXJ3, TCF7L2, and TCF7 as critical in the regulation of AT2 mitochondrial respiration. These findings provide candidate mitochondrial regulators for future studies to investigate the roles and mechanisms of mitochondrial regulatory factors in lung aging and age-related diseases.

## 2 Results

### 2.1 Expressions of classical senescence markers are not significantly changed in AT2 across different stages of adult aging

**FIGURE 1:**
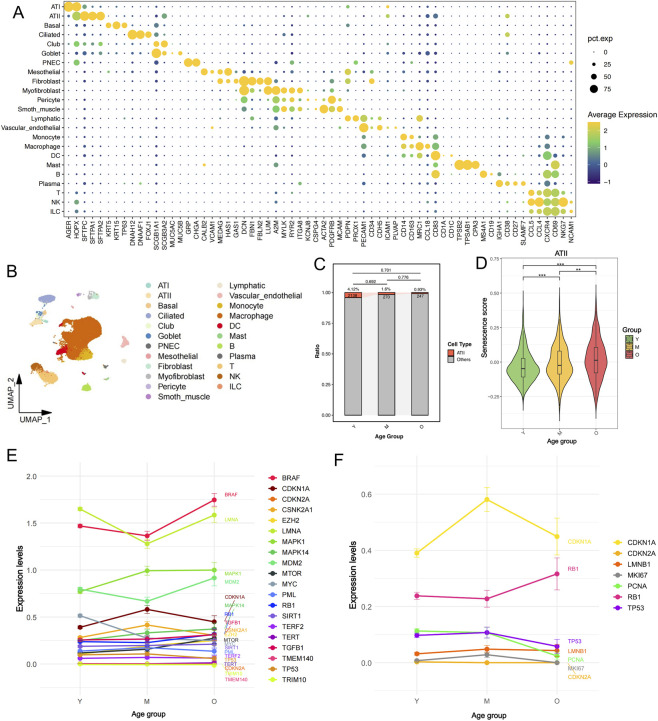
Single-cell RNA-seq analysis reveals an increased senescence score in aging AT2. **(A)** Dot plot illustrating the expression patterns of canonical marker genes (columns) across major lung-cell populations (rows). **(B)** Uniform Manifold Approximation and Projection (UMAP) representation of the 23 identified cell populations (95,301 cells). AT, alveolar type; PNEC, pulmonary neuroendocrine cell; DC, dendritic cell; NK, natural killer; ILC, innate lymphoid cell. **(C)** Statistical bar plot of the proportion of AT2 in each age group. **(D)** Violin plot visualization of the cellular senescence score based on the top 20 ranked senescence genes of the AT2, and the Wilcoxon test was performed among three groups. **(E)** Expression level of the top 20 genes in each age group. **(F)** Expression level of classical aging markers in each age group. **p < 0.01 and ***p < 0.001.

To characterize the transcriptomic changes in human AT2 across different stages of adult aging, we analyzed a publicly available single-cell RNA sequencing (scRNA-seq) dataset from 28 healthy lung donors (GSE136831), as described in *Methods*. The samples were divided into three age groups as described in a previous study (Xia et al., 2022): young (20–44 years; average age: 29 years), middle-aged (45–64 years; average age: 54.4 years), and old (≥65 years; average age: 68.3 years). The detailed information of each group is provided in Supplementary Table S1. After stringent quality control and normalization, a total of 95,301 cells were clustered into 23 distinct cell types, representing the major lung lineages: stromal, endothelial, epithelial, and immune lineages, based on canonical cell-type specific marker genes (Figure 1A). These cell types were visualized using Uniform Manifold Approximation and Projection (UMAP) (Figure 1B).

To investigate age-associated changes in AT2, we compared their proportions across different age groups. The highest proportion of AT2 was observed in the young group (Y: 4.12%, 2,138 per 51,843 cells), followed by the middle-aged (M: 1.60%, 270 per 16,864) and old (O: 0.93%, 247 per 26,594 cells) groups (Figure 1C), demonstrating a progressive decrease in AT2 cell abundance with increasing age. However, no statistical significance was identified (Y vs. M: p = 0.692; M vs. O: p = 0.776; Y vs. O: p = 0.701). To test whether AT2 senescence was progressively elevated with increasing age, we performed a comparison of cellular senescence scores using multiple previously reported methods. First, using a top-20 senescence-associated genes from the CSGene dataset (Zhao et al., 2016), the cellular senescence score was significantly elevated in the aged AT2 (Figure 1D). Among these genes, the expressions of EZH2, MAPK1, MDM2, MTOR, and BRAF were significantly increased in the old group, whereas MYC was markedly decreased (Figure 1E). Second, we performed two additional approaches, SENMAYO (Saul et al., 2022; Suresh et al., 2024) and hUSI (Wang et al., 2025), to further evaluate aging-associated features in AT2 cells. Neither method detected a significant senescence signal in AT2 cells (Supplementary Figure S1A, S1B). Third, we analyzed the expression level of classical senescence markers, including CDKN1A, CDKN2A, LMNB1, MKI67, PCNA, RB1, and TP53 (Supplementary Figure SC), and found no significant changes across different stages of adult aging (Figure 1F). These results suggest that aging-associated changes in AT2 cells of healthy individuals may not fully align well with the canonical senescence genes.

### 2.2 Reduced expression of mitochondrial respiration-related genes represents a defining feature during AT2 aging

**FIGURE 2:**
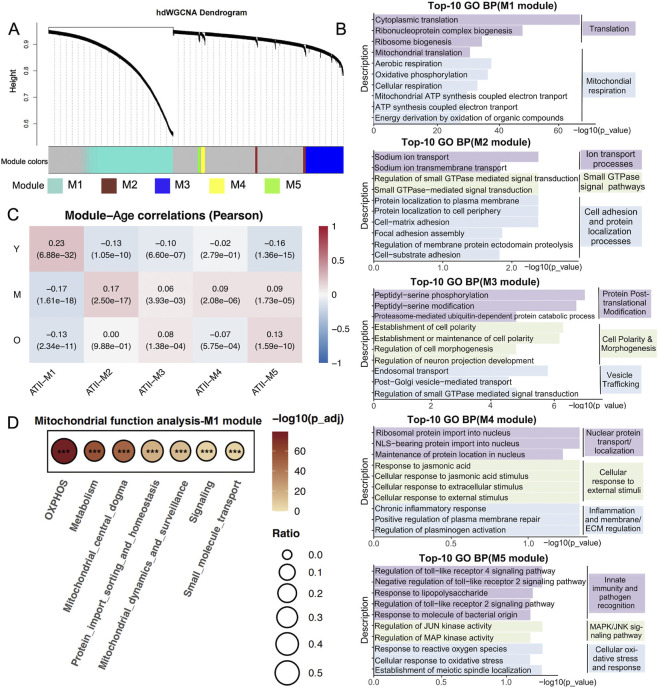
Transcriptional dysregulation of nuclear-encoded mitochondrial genes across functional modules in aging AT2. **(A)** Co-expression network computation and gene module detection is performed in one step using the function Construct Network. **(B)** The top 10 GO–BP pathway enrichment analysis of genes in each module. **(C)** Heatmap plot shows the correlation between each module and age group. **(D)** Functional classification of nuclear-encoded mitochondrial genes in the M1 module using the MitoCarta 3.0 database. ***p < 0.001.

To characterize age-associated transcription changes in AT2, we performed hdWGCNA, a framework optimized for sparse and noisy single-cell transcriptomes by aggregating cells into “metacells” and constructing cell-type-specific networks (Morabito et al., 2023). Using this approach, we constructed an AT2-specific expression network (soft threshold power β = 5 at scale-free topology *R* ^2^ = 0.80) (Supplementary Figure S2A) and identified five co-expression modules (M1 to M5) after batch correction with Harmony (Figure 2A; Supplementary Table S4). Module connectivity was assessed for each gene (Supplementary Figure S2B). The top 10 Gene Ontology–Biological Process (GO–BP) terms revealed distinct biological themes across modules (Figure 2B). Correlation analysis revealed that M1 exhibited the strongest association with aging, displaying a positive correlation, whereas the M5 module showed a positive correlation with age (Figure 2C). The M1 module was enriched for mitochondrial respiration, along with energy-demanding processes such as translation and M5 with innate immune responses, MAPK/JNK signaling, and oxidative stress. These results suggest that the AT2 aging program appears to be characterized by decreased OXPHOS, along with increased responsiveness to stress and immune-related signaling. Then, we annotated M1 genes with seven different mitochondrial-related functions according to MitoCarta 3.0 (Rath et al., 2021). The detailed gene lists for each functional module are provided in Supplementary Table S2. Consistent with the GO–BP analysis of the M1 genes (Figures 2B,D), this result revealed that OXPHOS, also known as mitochondrial respiration, was the most significantly changed category. In addition, GO–BP analysis of the top 5,000 expressed genes in AT2 cells demonstrated that core cellular processes such as translation and nuclear transport were enriched in all age groups. However, mitochondrial pathways, including cellular respiration, aerobic respiration, and oxidative phosphorylation, were predominantly enriched in young AT2 cells (Supplementary Figure S2C). Taken together, these findings demonstrate that the transcription program governing mitochondrial respiration in AT2 is progressively downregulated with aging.

### 2.3 Identification of transcription factors potentially programs mitochondrial function during AT2 aging

**FIGURE 3:**
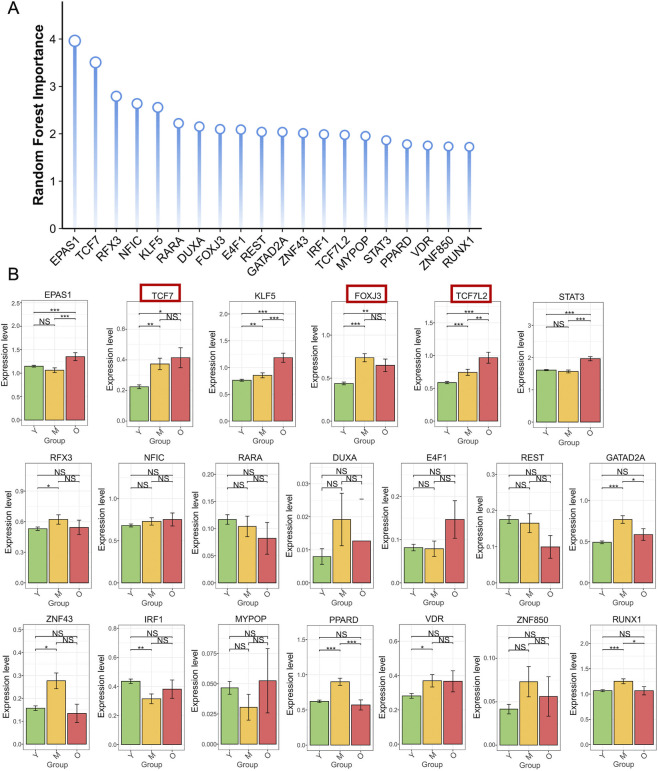
Age-associated transcription factors predicted to regulate NEMGs in AT2. **(A)** Candidate TFs regulating NEMGs in the M1 module were identified using a random forest-based feature selection approach and ranked using a random forest importance model, and the top 20 TFs were displayed according to their ranking. **(B)** The transcription level of each transcription factor in AT2 with aging. NS > 0.05, *p < 0.05, **p < 0.01, and ***p < 0.001.

To identify transcription factors (TFs) that are potentially responsible for the age-associated expression changes in nuclear-encoded mitochondrial genes (NEMGs), we used MEME suite (version 5.5.0) to infer gene co-expression networks and assess TF binding motif enrichment in promoter regions (2,200 bp upstream to 500 bp downstream of the TSS) of NEMGs within the M1 module. We then used a random forest-based algorithm ranking TFs by their regulatory importance and identified the top 20 candidates, namely, EPAS1, TCF7, RFX3, NFIC, KLF5, RARA, DUXA, FOXJ3, E4F1, REST, GATAD2A, ZNF43, IRF1, TCF7L2, MYPOP, STAT3, PPARD, VDR, ZNF850, and RUNX1 (Figure 3A). Among these TFs, only six TFs—EPAS1, TCF7, KLF5, FOXJ3, TCF7L2, and STAT3—displayed significant age-associated upregulation, whereas the others remained largely unchanged (Figure 3B). Among them, EPAS1, KLF5, and STAT3 are well-established regulators of mitochondrial function and homeostasis (Oktay et al., 2007; Mantel et al., 2012; Bao et al., 2021; Fang et al., 2024), whereas the roles of TCF7, FOXJ3, and TCF7L2 in mitochondrial function remain largely unclear. To explore whether the aging-associated expression changes in those TFs are conserved in species, we analyzed another published scRNA-seq dataset (GSA: CRA002577) (Ma et al., 2021) of young (4–6 years) and aged (18–21 years) cynomolgus monkeys, roughly corresponding to young adult (∼20 years) and elderly humans (∼70 years), respectively. Consistent with the human result, FOXJ3 and TCF7L2 were significantly upregulated in aged AT2 cells of cynomolgus monkeys, whereas changes in TCF7 expression was not detected (Supplementary Figure S3).

**FIGURE 4:**
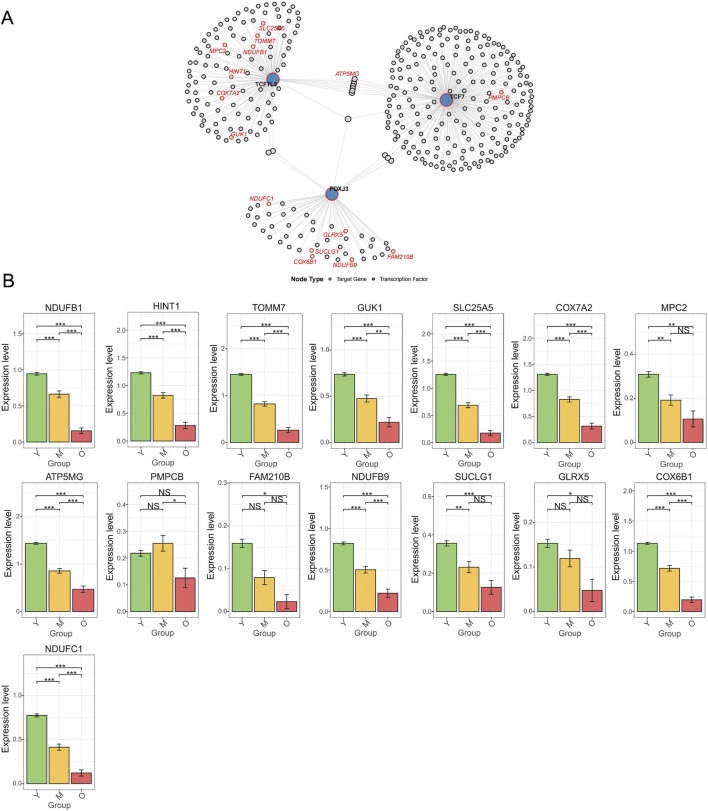
Expression level of target genes in AT2 during aging. **(A)** Gene regulatory network of TCF7, FOXJ3, and TCF7L2 in AT2 cells. Predicted downstream targets were identified based on TF-binding motifs (TCF7: 213; FOXJ3: 50; TCF7L2: 118). High-confidence interactions (p < 0.05) were retained, with target genes highlighted in red. **(B)** The transcription level of these candidate target genes in AT2 with aging. NS *p* > 0.05, *p < 0.05, **p < 0.01, and ***p < 0.001.

To predict how TCF7, FOXJ3, and TCF7L2 programs mitochondrial function during AT2 aging, we constructed a TF-gene regulatory network. Based on TF-binding motifs, TCF7L2 was predicted to have 118 putative downstream targets, FOXJ3 had 50, and TCF7 had 213 (Figure 4A). To increase the reliability of TF–target interactions, we retained only those with high-confidence interactions (p < 0.05) as candidate targets. Genes with high-confidence interactions (p < 0.05) were highlighted in the network, which are marked in red (Figure 4A). The TCF7L2 candidate targets, including NDUFB1, HINT1, TOMM7, GUK1, SLC25A5, COX7A2, MPC2, and ATP5MG, are mainly involved in ATP synthesis. TCF7 was predicted to regulate PMPCB (involved in mitochondrial protein processing) and ATP5MG (a complex V subunit), both central to energy production. The candidate targets of FOXJ3 include FAM210B, NDUFB9, SUCLG1, GLRX5, COX6B1, and NDUFC1, critical for mitochondrial energy production (Figure 4A). All of these target genes were consistently downregulated during AT2 aging (Figure 4B). Collectively, our data demonstrated an inverse expression relationship between TCF7L2, TCF7, and FOXJ3 and their downstream targets, suggesting that these TFs might act as negative regulators of mitochondrial respiration in AT2, a process involved in ATP production.

### 2.4 Experimental validation of TCF7L2, TCF7, and FOXJ3 function on mitochondrial respiration

**FIGURE 5:**
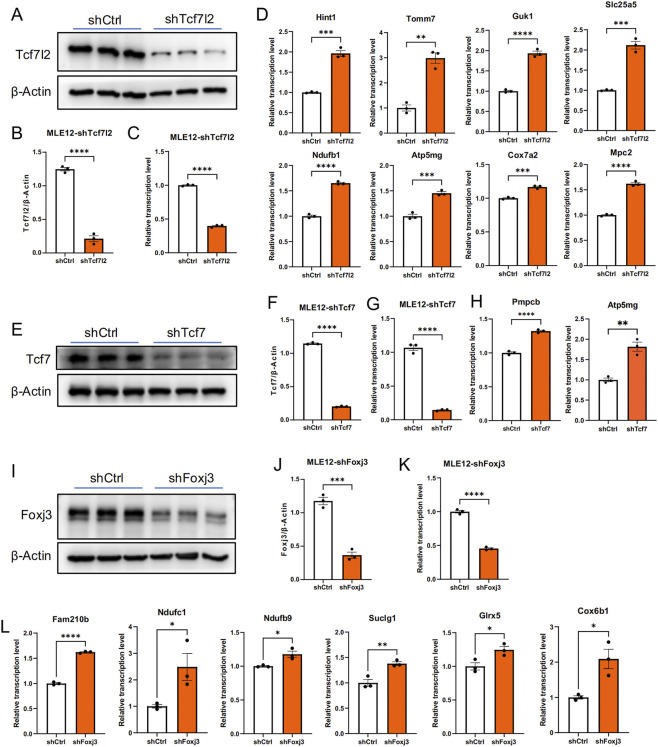
Effects of TCF7L2, TCF7, and FOXJ3 on their downstream target genes in MLE12 cells. **(A)** Validation of knockdown efficiency of Tcf7l2 by shTcf7l2 in MLE12 cells using Western blot. **(B)** Quantification of the Tcf7l2 protein level following shTcf7l2 knockdown. **(C)** RT-qPCR validation of Tcf7l2 knockdown by shTcf7l2. **(D)** RT-qPCR analysis of downstream target gene expression following Tcf7l2 knockdown. **(E)** Validation of knockdown efficiency of Tcf7 by shTcf7 in MLE12 cells using Western blot. **(F)** Quantification of the Tcf7 protein level following shTcf7 knockdown. **(G)** RT-qPCR validation of Tcf7 knockdown by shTcf7. **(H)** RT-qPCR analysis of downstream target gene expression following Tcf7 knockdown. **(I)** Validation of knockdown efficiency of Foxj3 by shFoxj3 in MLE12 cells using Western blot. **(J)** Quantification of the Foxj3 protein level following shFoxj3 knockdown. **(K)** RT-qPCR validation of Foxj3 knockdown by shTcf7l2. **(L)** RT-qPCR analysis of downstream target gene expression following Foxj3 knockdown. All experiments were conducted three times independently. *p < 0.05, **p < 0.01, ***p < 0.001, and ****p < 0.0001.

To validate whether TCF7L2, TCF7, and FOXJ3 inhibited the expression of their downstream genes, we designed gene-specific shRNAs to achieve targeted knockdown in MLE12 cells, an established mouse AT2 cell line. Western blot and qPCR analyses confirmed efficient knockdown of each TF at both protein and mRNA levels (Figures 5A–C,E–G, I–K). Knockdown of TCF7L2, TCF7, and FOXJ3 individually resulted in a significant upregulation of their downstream target genes in MLE12 cells (Figures 5D,H,L). Consistent results were obtained in A549 cells, a lung adenocarcinoma cell line widely used as a model of human alveolar epithelial cells (Supplementary Figure S4A-L). Only few expression discrepancies were observed, such as MPC2 upon TCF7L2 knockdown (Supplementary Figure S4D) and FAM210B following FOXJ3 knockdown (Supplementary Figure S4L). These inconsistencies may reflect the tumor-derived background of the A549 cell line. Overall, these results demonstrate that TCF7L2, TCF7, and FOXJ3 act as negative regulators of expression of NEMGs in AT2.

**FIGURE 6:**
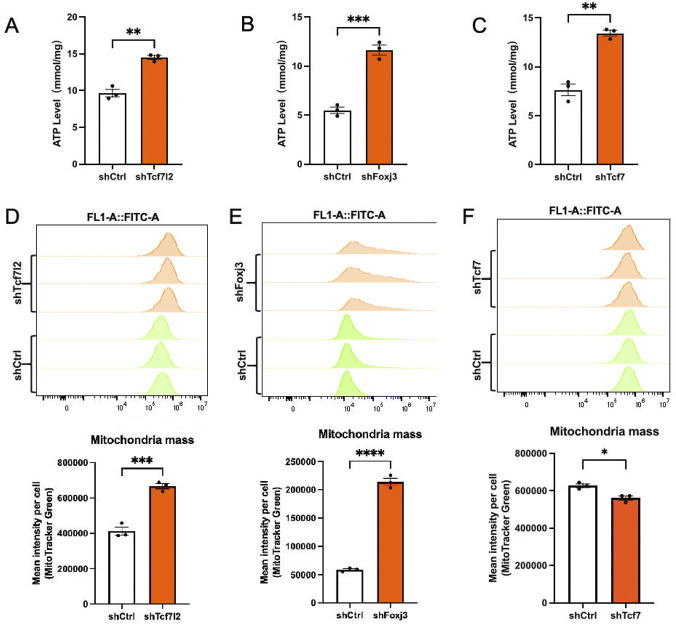
Effects of TCF7L2, FOXJ3, and TCF7 knockdown on ATP production. **(A–C)** The ATP level was measured in MLE12 cells following individual knockdown of TCF7L2, FOXJ3, and TCF7. **(D–F)** Total mitochondrial mass as determined with Mito Tracker Green in MLE12 cells after knockdown of TCF7L2, FOXJ3, and TCF7, respectively. Bar graph representation of data. All experiments were conducted three times independently. *p < 0.05, **p < 0.01, ***p < 0.001, and ****p < 0.0001.

To confirm the functions of TCF7L2, FOXJ3, and TCF7 on mitochondrial respiration in AT2, cellular ATP level and mitochondrial mass were compared between shCtrl and shTFs in alveolar-derived epithelial cells. The result showed that the ATP level was significantly increased after TCF7L2, FOXJ3, and TCF7 knockdown in MLE12 cells (Figures 6A–C). Consistent with these findings, knockdown of TCF7L2 and FOXJ3 also enhanced ATP production in A549 cells, whereas no changes in ATP levels were observed after TCF7 knockdown (Supplementary Figure S5A-F). In addition, an increased mitochondrial mass was identified in TCF7L2-knockdown and FOXJ3-knockdown MLE12 cells (Figures 6D,E), as indicated by an increase in the mean fluorescence intensity of MitoTracker Green. In contrast, TCF7 knockdown reduced mitochondrial mass, as reflected by a lower mean fluorescence intensity in MLE12 cells (Figure 6F). Taken together, these results consistently demonstrated that TCF7L2 and FOXJ3 act as negative transcriptional regulators of both ATP production and mitochondrial mass in AT2.

## 3 Discussion

The cellular and molecular hallmarks of aging, including genomic instability, telomere attrition, epigenetic alterations, loss of proteostasis, deregulated nutrient sensing, mitochondrial dysfunction, cellular senescence, stem cell exhaustion, and altered intercellular communication, are well established (Lopez-Otin et al., 2023). However, which hallmark plays the predominant role in governing AT2 cell aging remains undefined. In this study, we demonstrate that mitochondrial respiration decline is a defining feature of AT2 cell aging and uncover key transcription factors that orchestrate the regulation of mitochondrial respiration-related genes during this process.

The accumulation of senescent cells is a hallmark of organ aging and a key driver of age-related diseases (Lopez-Otin et al., 2023). In the lung, increased cellular senescence compromises regenerative capacity and may contribute to disease development (Liu Z. et al., 2024). Notably, aging mechanisms are highly cell-type-specific, underscoring the need to examine how distinct pulmonary cell populations respond to aging. Our analysis revealed a significant increase in cellular senescence scores in AT2 during natural aging, consistent with the previous transcriptomic analyses (Jia et al., 2023; Liu X. et al., 2024). This concordance underscores that AT2 cells are particularly susceptible to age-associated stress, and their progressive senescence likely impairs regenerative capacity while contributing to the impaired repair characteristic of the aged lung. However, classical cellular senescence markers (CDKN1A, CDKN2A, TP53, LAMB1, and PCNA) were not significant changed (Ogrodnik et al., 2024) in aging AT2, along with senescence-related gene signatures (SenMayo and hUSI). We propose the following possible explanations: first, SENMAYO and hUSI were developed based on datasets with substantial age differences (e.g., ∼30 vs. ∼70 years), whereas the age differences between groups in our study are relatively modest, particularly between the middle-aged (M) and old (O) groups (54.4 vs. 68.3 years). Second, it is well known that the rate of lung progenitor cell (AT2) turnover is low compared to other organs (Bowden, 1983; Han et al., 2023). This low turn over time, combined with lifelong exposure to environmental stressors and cumulative oxidative and inflammatory insult, contributes to distinct aging characteristics of AT2 in the lung. The global gene set-based scoring approaches developed by SENMAYO and hUSI were based on data collected from bone marrow, brain, retina, prostate, and other diseased tissues. Therefore, it may not be well suited for assessing AT2 aging in healthy lung tissue. Interestingly, we identified impaired mitochondrial respiration as a central feature of AT2 during natural aging. As cellular powerhouses, mitochondria maintain energetic homeostasis, regulate oxidative stress, and modulate apoptosis. Mitochondrial dysfunction is tightly correlated with aging (Miwa et al., 2022). Age-related mitochondrial decline manifests across multiple biological processes, including impaired OXPHOS, elevated reactive oxygen species (ROS), altered mitochondrial dynamics, and compromised mitophagy (Lesnefsky and Hoppel, 2006; Sebastian et al., 2017; Guo et al., 2023; Adlimoghaddam et al., 2025). AT2 cells require exceptionally high energy to sustain surfactant synthesis and secretion, rendering them highly dependent on mitochondrial integrity and bioenergetic capacity. This is why AT2 cells are particularly vulnerable to mitochondrial dysfunction during aging. Although mitochondrial respiration defects in AT2 have been reported in previous studies in the context of age-related pulmonary diseases (Bueno et al., 2015; Yu et al., 2018), their contribution to physiological aging and roles in AT2 cell senescence remain unclear. Our discovery provides direct evidence that mitochondrial respiration dysfunction is not merely a consequence of disease but an intrinsic feature of natural AT2 cell aging. In this study, we further analyzed the expression of genes involved in other energy-producing pathways, including glycolysis and the TCA cycle. As shown in Supplementary Figure S3B, neither pathway exhibited significant changes in AT2 cells during aging. The observed reduction in OXPHOS, without a compensatory increase in glycolysis, suggests that aging AT2 cells may be unable to maintain metabolic demands through these pathways. These genes’ expression patterns in aged AT2 cells indicate a decreased energy production capacity, consistent with previous reports showing a progressive functional decline of AT2 cells with age.

Mitochondrial respiration is defined as the process by which mitochondria synthesize ATP through oxidative phosphorylation (Salmonowicz and Szczepanowska, 2025). Here, we identified an age-associated module—M1 module—that negatively correlates with aging and is enriched for genes involved in mitochondrial respiration, while M5, which is mainly enriched for oxidative stress and innate immune-related pathways, exhibits an age-associated trend opposite to that of M1. Notably, DUSP16, a negative regulator of MAPK/JNK signaling, is downregulated in middle-aged samples, suggesting a potential release of inhibitory control and increased sensitivity of stress-related pathways. In parallel, genes such as MYH9 and CD44 are upregulated, indicating the activation of cytoskeletal remodeling and cellular stress response processes. Together, these findings suggest that AT2 cells may shift toward a stress- and immune-responsive transcriptional state during aging, consistent with previous reports (Han et al., 2023; Quan et al., 2024). In summary, our data demonstrated that the AT2 aging program in healthy individual appears to be characterized by decreased OXPHOS, along with increased responsiveness to stress and immune-related signaling.

Pharmacological inhibition and genetic loss of function of the mitochondrial respiration can lead to premature senescence in other cell types (Yoon et al., 2003; Stockl et al., 2006; Moiseeva et al., 2009). Recent research suggests that multiple mitochondrial energy metabolism-related genes may serve as highly accurate biomarkers for diagnosing IPF (Yao et al., 2025). This suggests that impaired mitochondrial respiration may be associated with AT2 cell aging and may contribute to age-related lung disease progression. Based on the M1 module, we further identified three novel transcription factors—TCF7L2, FOXJ3, and TCF7—whose expression increased in aging AT2 and can negatively regulate the expression of mitochondrial respiration-related genes. In another study, TCF7L2 has been shown to regulate PGC-1α expression, thereby regulating mitochondrial biogenesis and OXPHOS activity, ultimately controlling stem cell differentiation (Wanet et al., 2017). FOXJ3 has been implicated in mitochondrial biogenesis through miRNA-mediated regulation in cancer and muscle differentiation (Yamamoto et al., 2012; Barisciano et al., 2021); however, how FOXJ3 itself governs mitochondrial biogenesis has not been specifically investigated. TCF7 is mainly involved in T-cell development and differentiation (Zhu et al., 2015). Knockdown of TCF7 decreased glycolytic capacity and mitochondrial respiratory function, thereby impairing T-cell proliferative capacity (Cai et al., 2022). Our study provides the first evidence that mitochondrial respiration-related genes, including NDUFB1, COX7A2, and ATP5MG, are TCF7L2 target genes; NDUFC1, NDUFB9, and COX6B1 are regulated by FOXJ3; ATP5MG is also a target of TCF7. Inhibition of TCF7L2 and FOXJ3 increased both ATP production and mitochondrial mass; in contrast, although TCF7 deficiency enhanced ATP production, it led to a decrease in mitochondrial mass. This discrepancy could be explained by the fact that ATP production is primarily controlled by mitochondrial OXPHOS, specifically the electron transport chain (ETC) and ATP synthase activity. In contrast, mitochondrial mass is maintained by a distinct set of processes, including mitochondrial biogenesis, fusion/fission dynamics, and mitophagy. Consequently, the former emphasizes energy transduction efficiency, while the latter ensures mitochondrial quantity and structural integrity. TCF7 predominantly regulates genes associated with mitochondrial protein processing (PMPCB) and ATP synthase (ATP5MG). These targets are critical for energy conversion efficiency of mitochondria but are not directly linked to mitochondrial mass. Consequently, TCF7 knockdown likely enhances the functional output of existing mitochondria, leading to increased ATP production without a corresponding increase in mitochondrial mass, and may even slightly reduce organelle abundance through adaptive quality control mechanisms. In contrast, the downstream targets of FOXJ3 and TCF7L2 include multiple genes involved in mitochondrial respiration and organelle maintenance (e.g., FAM210B, NDUFB9, SUCLG1, GLRX5, COX6B1, NDUFC1, NDUFB1, HINT1, TOMM7, GUK1, SLC25A5, COX7A2, MPC2, and ATP5MG). These factors collectively influence both ATP production and mitochondrial mass. The discovery of these transcription factors provides mechanistic insight into the molecular regulatory drivers of mitochondrial respiration dysfunction during AT2 cell aging.

In summary, we demonstrate that mitochondrial respiration decline is a defining feature of AT2 cell aging and uncover key transcription factors (TCF7L2, FOXJ3, and TCF7) that regulate mitochondrial respiration-related genes during this process. However, several limitations should be acknowledged. First, functional validation of the identified transcription factors was performed using established alveolar epithelial cell lines, which may not fully recapitulate the physiological and metabolic characteristics of primary AT2 cells, potentially introducing cell line-specific biases. Second, the scRNA-seq analysis was based on a limited number of individuals, with only one sample representing the population aged above 70, which may introduce selection bias and limit the generalizability of aging-associated findings. Future studies will incorporate primary human AT2 cells, organoid systems, and *in vivo* models for functional validation and mechanistic exploration, along with larger, well-balanced cohorts across age groups, to more comprehensively characterize the transcriptional and metabolic dynamics of AT2 cells in the process of aging and development of age-related pulmonary diseases.

## 4 Materials and methods

### 4.1 Data acquisition and preprocessing

Single-cell RNA sequencing (scRNA-seq) data (10X Genomics) of lungs from 28 healthy individuals were collected from Adams et al. (2020) (GEO accession numbers: GSE136831). These individuals were aged 20-80 years and classified into three groups: young (20-44 years), middle-aged (45-64 years), and old (≥65 years), consistent with the original method of the article from which the dataset was derived. Data processing and analyses were performed using the Seurat R package (version 4.1.0) with default parameters unless otherwise specified. Then, quality control was performed by retaining cells with nUMI >1000 and <20% mitochondrial transcripts. LogNormalize was conducted by setting scale.factor = 10000, and FindVariableFeatures was performed using the vst method to generate 3,000 highly variable genes for PCA-based dimension reduction. The top 30 principal components were used for clustering via the Louvain algorithm at a resolution of 0.8. Cluster identities were assigned based on canonical marker genes and compared to the original annotations for validation.

### 4.2 Senescence score analysis

To compare the senescence score among young, middle-aged, and old groups, we collected the top 20 ranked senescence genes (CSGene) based on their frequency in the literature (Zhao et al., 2016) and calculated the module scores using the AddModuleScore function in Seurat. Then, the wilcox.test was performed among three groups (0.01 < *p* < 0.05 was marked by *, 0.001 < *p* < 0.01 was marked by **, and *p* < 0.001 was marked by ***).

### 4.3 Functional enrichment comparison of AT2 cells across age groups

To investigate age-associated variations in gene function of AT2 cells, we performed AverageExpression to obtain the gene expression for three groups separately. Then, the top 5,000 genes were extracted for Gene Ontology (GO) enrichment analysis, including biological processes (BP), cellular components (CC), and molecular functions (MF), using the clusterProfiler package (Wu et al., 2021) (version 4.6.2) in R, with the human gene annotation database org.Hs.eg.db (version 3.16.0) as the reference. Default parameters were applied throughout, and terms or pathways with an adjusted p-value <0.05 were considered significantly enriched. In addition, the top 15 GO–BP terms for these three groups were compared and plotted in a bubble plot.

### 4.4 Gene module identification using hdWGCNA

To identify gene co-expression modules of AT2 within scRNA-seq data, we employed the hdWGCNA R package (version 0.4.05), which is tailored for high-dimensional transcriptomic analyses (Morabito et al., 2023). We prepared the Seurat object with SetupForWGCNA function and selected genes expressed in at least 5% of cells (gene_select = “fraction,” fraction = 0.05), ensuring the inclusion of genes with sufficient expression levels across the dataset. To mitigate sparsity and improve the robustness of co-expression network construction, we aggregated similar cells into metacells using the MetacellsByGroups function, grouping cells based on shared sample (group.by = “Sample”), applying a k-nearest neighbors (kNN) approach with k = 10, allowing overlap (max_shared = 15) and enforcing a minimum of 10 cells per metacell. Subsequently, the metacell expression matrix was normalized using NormalizeMetacells and set for co-expression network analysis using SetDatExpr. We evaluated soft-thresholding powers with TestSoftPowers and visualized candidates using PlotSoftPowers; based on the scale-free topology fit index and mean connectivity, we chose soft_power = 5. Using this power, we constructed the weighted gene co-expression network with ConstructNetwork, computed and stored the topological overlap matrix (TOM), derived module eigengenes with ModuleEigengenes (first principal component of each module), and assessed within-module connectivity with ModuleConnectivity. Finally, we generated a dendrogram to depict the hierarchical clustering of modules.

### 4.5 Chartered comparison of gene modules

The five identified gene modules were correlated to the Y, M, and O groups using the Pearson correlation test, and the correlation coefficients and corresponding P-values were visualized in a heatmap. Then, to perform a comprehensive comparison of gene modules, we conducted functional enrichment analyses for each module using Gene Ontology and Kyoto Encyclopedia of Genes and Genomes pathways, following the same methodology described earlier. Additionally, we examined the mitochondrial-related gene composition of module 1 using human reference genes from the MitoCarta3.0 database (https://www.broadinstitute.org/mitocarta) (Rath et al., 2021), which catalogs 1,138 human mitochondrial genes. These genes are associated with various mitochondrial functions, including 1) oxidative phosphorylation (OXPHOS), 2) mitochondrial central dogma, 3) protein import sorting and homeostasis, 4) mitochondrial dynamics and surveillance, 5) signaling, 6) metabolism, 7) small-molecule transport. For this, we classified module-classified genes into seven categories based on each functional role, and the chi-square tests were performed to determine the significant distribution of genes across the categories. (0.01 < *p* < 0.05 was marked by *, 0.001 < *p* < 0.01 was marked by **, and *p* < 0.001 was marked by ***).

### 4.6 Prediction of transcript factors

To predict potential transcription factors regulating NEMGs in the M1 module, promoter sequences of all NEMGs were first extracted using a custom Python script. Promoter regions were defined as the region spanning from 2,200 bp upstream to 500 bp downstream of the transcription start site (TSS). High-confidence position weight matrices (PWMs) for human transcription factors were obtained from the HOCOMOCO v13 database (H13CORE, available at https://hocomoco13.autosome.org/) in MEME format. The database was preprocessed to remove redundant motifs and ensure compatibility with MAST, and both DNA strands were considered during analysis. Promoter sequences were scanned using the Motif Alignment and Search Tool (MAST) from the MEME suite (v5.5.0) (Bailey et al., 2009). MAST was chosen for its efficiency in identifying potential transcription factor binding sites using PWMs and for providing statistical significance scores that allow robust comparison across large sets of sequences. During scanning, low-confidence matches were filtered using the default p-value threshold (0.0001); adjustments were made for sequence composition, and only the best non-overlapping match per motif was reported to simplify downstream analysis. For each identified site, MAST calculates a match score by summing the frequencies of nucleotides at each position according to the PWM. This approach, combining a large, high-confidence motif database with efficient scanning and scoring, enabled sensitive and specific identification of candidate transcription factors potentially regulating Module 1 genes. The resulting ranked motif list was used for further analysis.

### 4.7 Identification of key transcription factors and GRN construction

Key TFs regulating NEMGs in the M1 module were identified using a random forest-based feature selection approach (Basu et al., 2018). TFs with significant correlation to NEMG expression (Pearson’s p < 0.05) were first retained and then used as input features in a random forest model, with NEMGs expression as the response variable. TF importance scores derived from the model were used to rank all candidates, and the top 20 TFs were selected as key regulators. This strategy combines correlation analysis with machine learning-based feature importance, enabling robust identification of TFs that are both strongly associated with and likely to regulate NEMGs.

To construct the gene regulatory network (GRN), the selected TFs were paired with their predicted target genes within the M1 module. Interactions between TFs and target genes were visualized using edges, and only high-confidence interactions, defined by significant correlation thresholds as applied previously, were highlighted in the network. This visualization provides a clear representation of potential regulatory relationships and facilitates the identification of hub TFs and target genes within the module.

### 4.8 Cell culture

The MLE12 cell line (Immocell, #IM-M015) and A549 cell line (CSTR:16607.09.1101HUM-PUMC000011, the National Biomedical Cell-Line Resource, NSTI-BMCR) were used as mouse and human alveolar Type II epithelial cell models, respectively. MLE12 cells were maintained in RPMI 1640 (GIBCO Cat. #430-1800 EA with glutamine and without sodium bicarbonate), with 5 μg/mL transferrin (Sigma Cat. #T-8027, Bovine), 0.1 nM hydrocortisone (Sigma Cat. #H-0888), 0.1 nM β-estradiol (Sigma Cat. #E−2758), 0.01 M L-glutaMAX (Sigma Cat. #G-7513), 0.01 M HEPES (Sigma Cat. #H-0887), penicillin/streptomycin, and 1X insulin–transferrin–sodium selenite (Sigma Cat. #l-1884), supplemented with 5% FBS (BioInd Cat. #04-001-1A). A549 cells were maintained in RPMI 1640, with 10% FBS and 1% penicillin/streptomycin. All cell lines were maintained in a 37 °C incubator with 5% CO2.

### 4.9 Plasmid construction, viral production, and cell infection or transfection

For functional knockdown of TCF7L2/Tcf7l2, FOXJ3/Foxj3, and TCF7/Tcf7, an shRNA-based approach was used. The sequences of shRNAs against human TCF7L2, FOXJ3, and TCF7, along with mouse Tcf7l2, Foxj3, and Tcf7, were the validated MISSION shRNAs (Sigma-Aldrich). The shRNAs were TRCN0000262847 (human TCF7L2), TRCN0000012180 (mouse Tcf7l2), TRCN0000020802 (human FOXJ3), TRCN0000082210 (mouse Foxj3), TRCN0000021674 (human TCF7), and TRCN0000360415 (mouse Tcf7) and subcloned to the lentiviral vector pLKO.1. Viral production and cell infection were performed essentially as described (Chen et al., 2021). For stable knockdown assays, virus packaging plasmids and shRNA constructs were transfected into HEK293T cells using polyethylenimine (PEI). After 48 h of transfection, the culture supernatant was collected and mixed with the virus concentration solution, followed by overnight incubation at 4 °C. The mixture was then centrifuged at 3,000 rpm at 4 °C to concentrate lentiviral particles for subsequent cell infection. After 48 h of infection, cells were selected with 2 μg/mL puromycin for 3 days. The cells were then further cultured until evident phenotypic changes were observed or harvested for other assays. Virus packaging with the empty vector and cell infection with the virus were performed in parallel, which was used as a negative control for the knockdown assay.

### 4.10 Immunoblotting

Whole-cell lysates (WCLs) were prepared, and IB detection of protein expression in cells was carried out using conventional methods. A total of 25 μg protein per lane was separated using SDS–PAGE and transferred onto PVDF membranes. Primary antibodies included β-ACTIN (Cell Signaling Technology, #4970, 1:5000), TCF7L2 (Abclonal, #A19548, 1:1000), FOXJ3 (Affinity, #AF0602, 1:1000), and TCF7 (HUABIO, #HA723582, 1:1000). The secondary antibodies were HRP-conjugated goat anti-rabbit or anti-mouse IgG (Sangon Biotech, #D110058; #D110087, 1:5000). Blots were developed with a Western blotting substrate (Tanon, #180–501). Blotting signals were quantified using ImageJ software.

### 4.11 Total RNA preparation and reverse transcriptase–quantitative polymerase chain reaction

Total RNAs were extracted from cells with TRIzol reagent (15596-026, Invitrogen) following the manufacturer’s instructions. cDNAs were transcribed from the total RNAs (1 μg per reaction) with the HiScript III RT SuperMix for qPCR (R323-01, Vazyme), which contains the reagent for cleaning up the contamination of genomic DNA. The resulting cDNA was diluted five-fold, and 1 μL of diluted cDNA was used for each qPCR reaction. qPCR was performed on a CFX96™ Real-Time system (BioRad) using the following parameters: one cycle of pre-denaturation at 95 °C for 5 min, followed by 40 cycles of denaturation at 95 °C for 10 s, annealing and extension at 60 °C for 30 s, and an additional cycle for melting the curve. Relative gene expression levels were calculated using the 2^-ΔΔCt^ method, with β-ACTIN or GAPDH as internal controls. Significance of changes in transcription was calculated based on experiments in triplicate using the unpaired Student’s t-test. Data were presented as histograms with relative units of transcription levels. Primers for RT-qPCR are listed in Supplementary Table S3.

### 4.12 Mitochondria mass assay

Mitochondria mass was detected using MitoTracker Green (Thermo Fisher M7514). According to the instructions, the probe was dissolved in 10 μM of DMSO and then diluted with RPMI 1640 into 30 nM for MLE12 and 200 nM for A549 cells; the cells were incubated at 37 °C for 30 min and then washed twice with 1× PBS (Servicebio G4207). All cell lines were responded in PBS after digestion of 0.25% Trypsin–EDTA. Fluorescence intensity was detected in FITC-A using CytoFLEX (Beckman).

### 4.13 Total ATP assay

Intracellular ATP levels were measured using the Enhanced ATP assay Kit (S0027, Beyotime). According to the instructions, detecting working solution was added onto a black 96-well plate and incubated for 5 min at room temperature. Lysis solution was added to cells with thorough mixing and centrifuged at 14,000 g for 10 min at 4 °C. The supernatant was added to the dark plate and mixed fast, followed by detection of the luminescence signals using a multimode reader (PE EnVision 2105). Total ATP levels were calculated on the basis of the luminescence signals accordingly. The supernatant was subjected to protein quantification analysis using an enhanced BCA protein assay kit (P0009, Beyotime), and the total ATP content was normalized based on the BCA results.

### 4.14 Statistical analysis

Statistical analyses were performed using GraphPad Prism software. Data were presented as the mean ± standard error of mean (SEM). Student’s t-test was used to analyze differences in protein and mRNA levels for a number of molecules between the control and experimental group. Significance levels are indicated by *p*-values: *p < 0.05, **p < 0.01, ***p < 0.001, ****p < 0.0001, and ^ns^ *p*>0.05.

## Data Availability

Publicly available datasets were analyzed in this study. These data can be found at: The scRNA-seq dataset analyzed in this study is publicly available in the GEO database under accession number GSE136831 and was originally published by Kaminski et al. (2020) [PMID: 32832599].
