# Supplementary material for: Aging-associated transcriptional programming of mitochondrial respiration in alveolar type II epithelial cells
Source: Front Cell Dev Biol. 2026 Jun 11;14:1740661. doi: 10.3389/fcell.2026.1740661 (PMC13293947; doi:10.3389/fcell.2026.1740661)
Supplement: Supplementary file 1 [file Supplementaryfile1.zip › Data Sheet 1.docx]

**Supplementary_Material**

**Aging Associated Transcriptional Programming of Mitochondrial Respiration in ATⅡ Cells**

**Lu Chen^1a^, Silu Hu^1a^, Xiaoju Tang^1a^, Yujun Wang^b^, Yin Zou^c^, Fengming Luo*^a^, Huajing Wan*^a^**


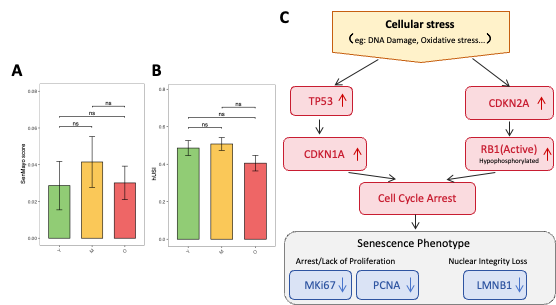


**Figure S1 Senescence-associated features in AT2 and Schematic illustration of the selection basis for classical senescence biomarkers.** A. Senescence scores calculated using the SENMAYO method across different age groups (Y, M, O) at the sample level. B. Senescence scores calculated using the hUSI method across different age groups (Y, M, O) at the sample level. Statistical comparisons were performed by pairwise Wilcoxon rank-sum tests with Benjamini–Hochberg correction. C. The classical senescence biomarkers used in this study were selected based on their established roles in canonical cellular senescence pathways. CDKN1A (p21) and CDKN2A (p16) represent key mediators of senescence-associated cell-cycle arrest. LMNB1 loss reflects senescence-related nuclear lamina remodeling. MKI67 and PCNA are proliferation-associated markers whose downregulation indicates irreversible growth arrest. RB1 and TP53 serve as central regulators of the p16–RB and p53–p21 pathways, respectively.


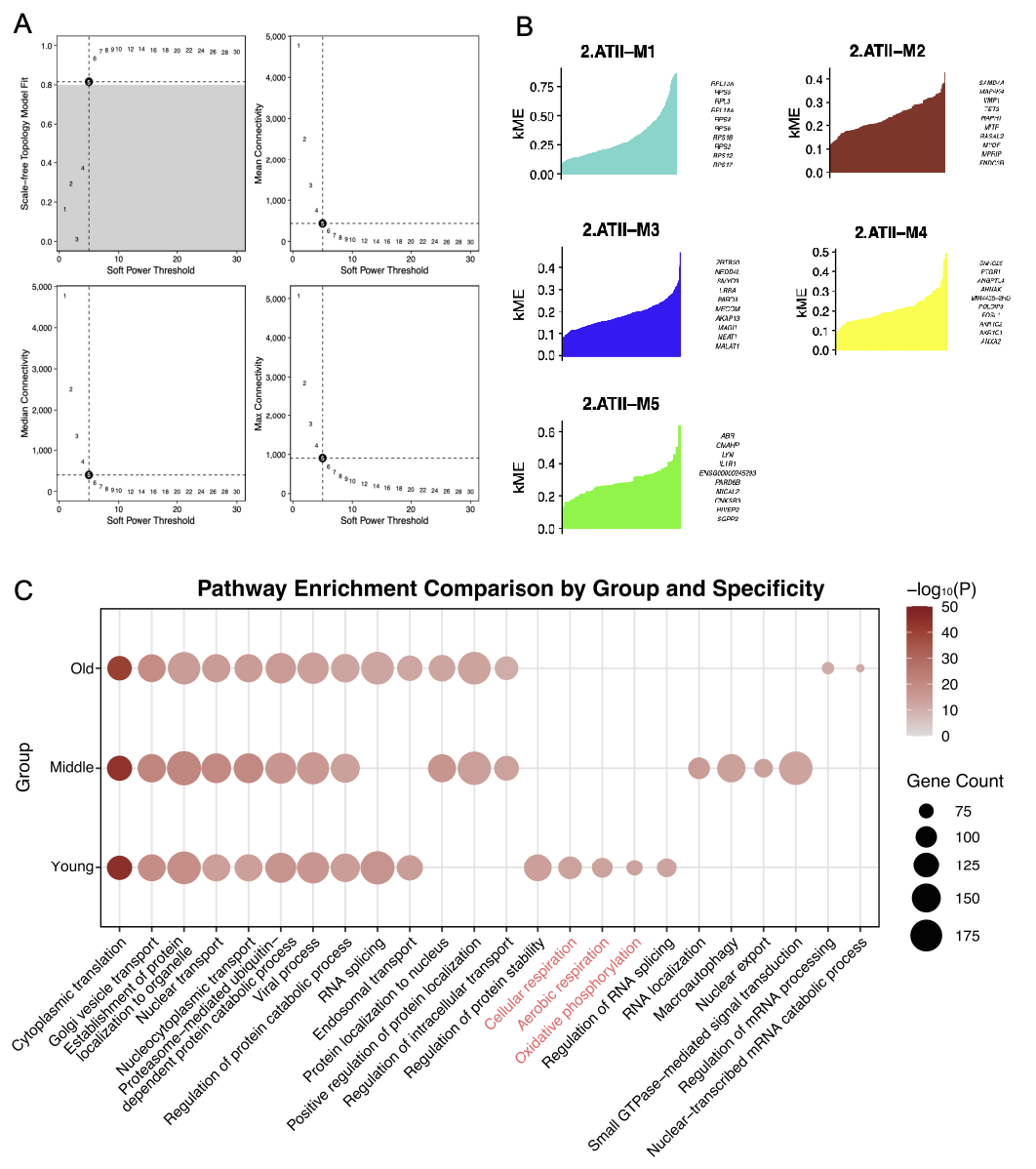


**Figure S2 Analysis of single-cell RNA sequencing data on human lungs from healthy subjects** A.Different values for the soft-power threshold β are tested using the functions TestSoftPowers and TestSoftPowersConsensus. B. Eigengene-based connectivity (kME) is computed for each gene using the ModuleConnectivity function. C. The characteristics of AT2 in different age groups were analyzed by biological processes pathway enrichment analysis.


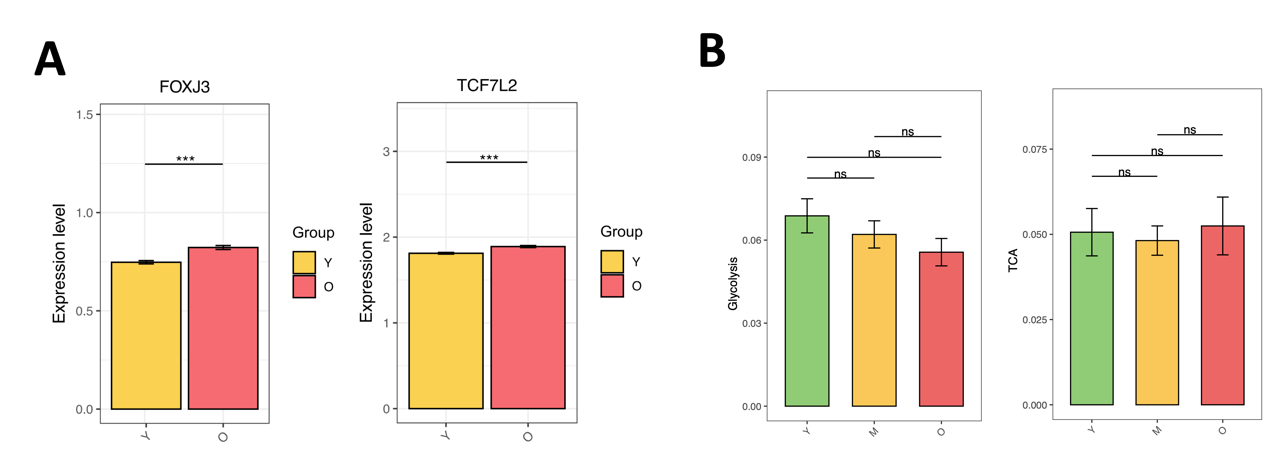


**Figure S3 Independent validation of FOXJ3 and TCF7L2 expression and metabolic features in AT2 cells.** A. Expression of FOXJ3 and TCF7L2 in AT2 cells from young and aged cynomolgus monkeys. This independent dataset was used to validate the age-associated expression trends of the identified transcription factors. B. Bar plots showing Glycolysis and TCA across age groups in AT2 cells, which was calculated using KEGG pathway gene sets using msigdbr (an R packages), and bars indicate mean ± SEM. Statistical comparisons were performed using the pairwise Wilcoxon rank-sum tests with Benjamini–Hochberg correction.


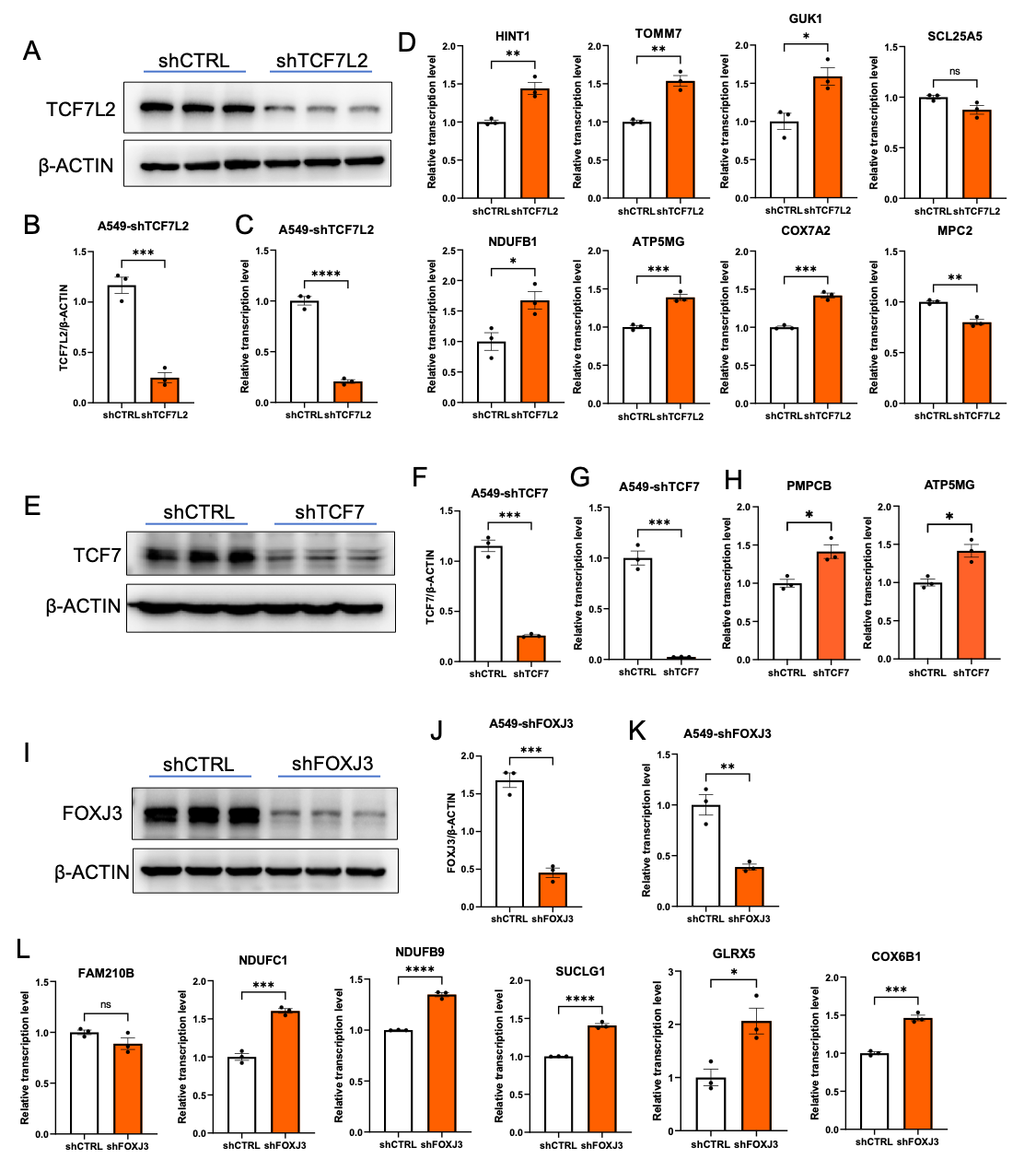


**Figure S4 Effect of TCF7L2, TCF7 and FOXJ3 on their downtream target genes in A549 cells** A. Validation of knockdown efficiency of TCF7L2 by shTCF7L2 in A549 cells using Western blot. B. Quantification of TCF7L2 protein level following shTCF7L2 knockdown. C. qPCR validation of TCF7L2 knockdown by shTCF7L2. D. qPCR analysis of downstream target gene expression following TCF7L2 knockdown. E. Validation of knockdown efficiency of TCF7 by shTCF7 in A549 cells using Western blot. F. Quantification of TCF7 protein level following shTcf7 knockdown. G. qPCR validation of TCF7 knockdown by shTCF7. H. qPCR analysis of downstream target gene expression following TCF7 knockdown. I. Validation of knockdown efficiency of FOXJ3 by shFOXJ3 in A549 cells using Western blot. J. Quantification of FOXJ3 protein level following shFOXJ3 knockdown. K. qPCR validation of FOXJ3 knockdown by shFOXJ3. L. qPCR analysis of downstream target gene expression following FOXJ3 knockdown. All experiments were conducted three times independently. ns p> 0.05, *p<0.05, **p<0.01, ***p<0.001, ****p<0.0001.


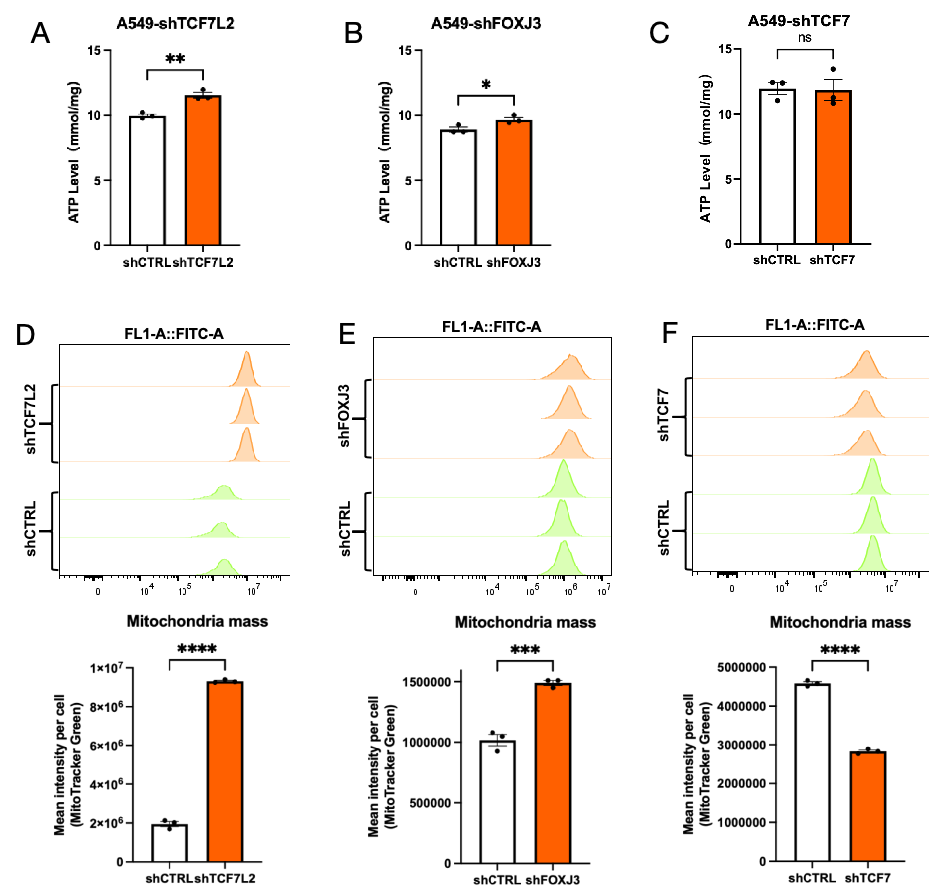


**Figure S5** **Effect of TCF7L2, FOXJ3 and TCF7 knockdown on ATP production in A549 cells** A-C. ATP level was measured in A549 cells following individual knockdown of TCF7L2, FOXJ3, and TCF7. D-F. Total mitochondrial mass as determined with Mito Tracker Green in A549 cells after knockdown of TCF7L2, FOXJ3, and TCF7, respectively. Bar graph representation of data. All experiments were conducted three times independently. ns p>0.05, **p<0.01, ***p<0.001, ****p<0.0001.

**Table S1 Characteristics of healthy lung samples used for cluster annotation.** Further analyses were conducted in Young (20-44 years), Middle-Aged (45-64 years) and Old (≥65 years) groups.


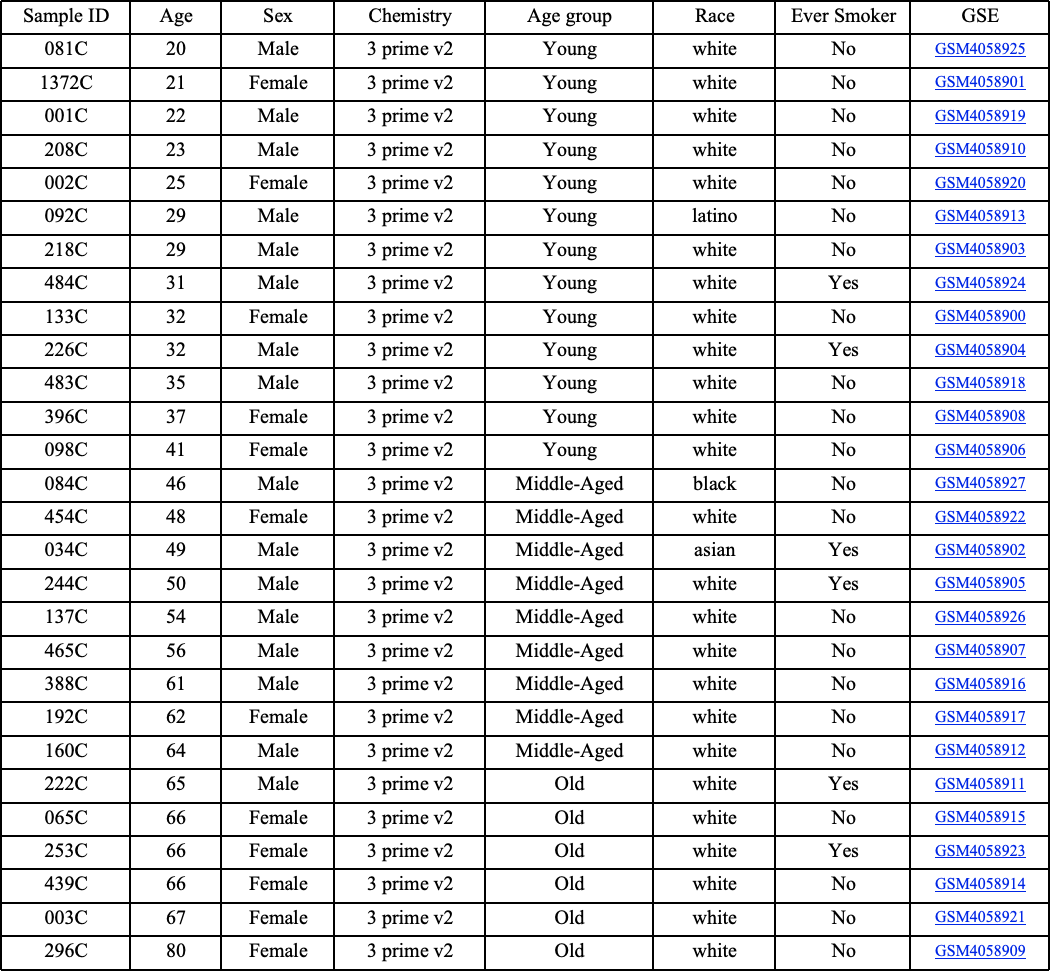


**Table S2 Distribution of M1 module NEMGs across the seven functional categories defined by MitoCarta 3.0.** NEMGs: nuclear-encoded mitochondrial genes.


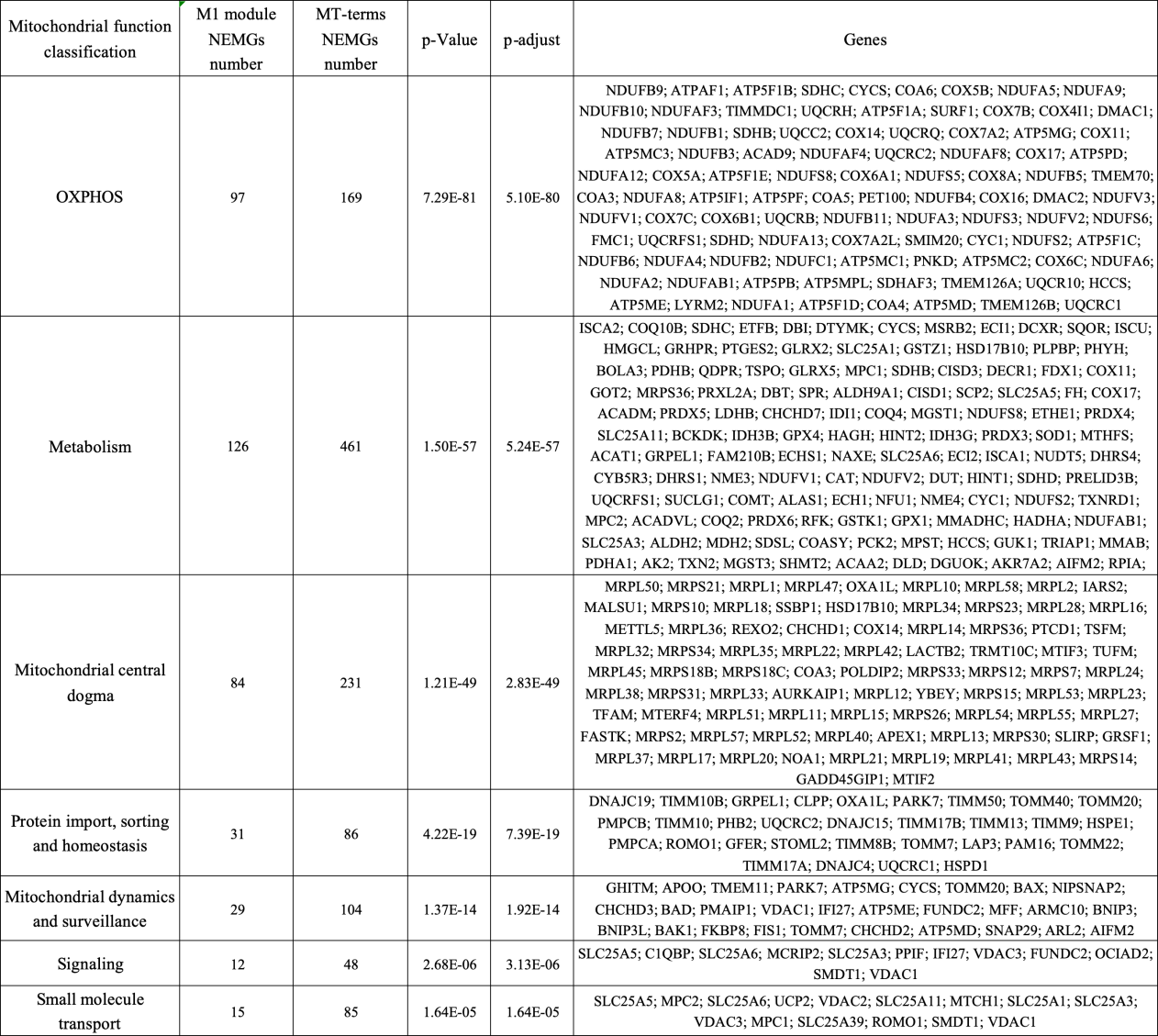


**Table S3 List of genes and their corresponding primers in the qPCR.**

| Target | Primer Sequences (5’ to 3’) |
| --- | --- |
| Tcf7l2 | Forword primer:ccaagaggcaagatggaggg Reverse primer:gggtttgtctgctctggagg |
| Foxj3 | Forword primer:gcctcggtccaaggatgatc Reverse primer:gagttggagaggcacttccc |
| Tcf7 | Forword primer:gacagctcccccatactgtg Reverse primer:caggtacaccagatcccagc |
| Hint1 | Forword primer:tttcccctcaagcaccaaca Reverse primer:tgcaccttcattcaccacca |
| Tomm7 | Forword primer:gagcaaagaagccaaacagagg Reverse primer:ccccaaagtaggcttaaaaccg |
| Guk1 | Forword primer:agcaggggacttcattgagc Reverse primer:gagggaggctgcacaaagat |
| Slc25a5 | Forword primer:agggcatcatagactgcgtg Reverse primer:ctgggtcctcttgtccacac |
| Nudfb1 | Forword primer:ttcagcttctacgtgagcactg Reverse primer:cttcttcattgggcctcagttc |
| Atp5mg | Forword primer:tgccgtgacttactcgaagc Reverse primer:gccaccaaaccattcagcac |
| Cox7a2 | Forword primer:ggaccatcagcaccacttca Reverse primer:caagcgtcagagccattgtg |
| Mpc2 | Forword primer:aatggggattggtgtgtgct Reverse primer:cctgctgaccccacaaagaa |
| Pmpcb | Forword primer:cgatctgtctcatggtcgca Reverse primer:caaccgtggcttgttcacag |
| Fam210b | Forword primer:ttttccaagagtacggggcc Reverse primer:gccattttggactgcaccag |
| Ndufc1 | Forword primer:cgtagtgctgcgctcgtt Reverse primer:atgaaaaccgaggcgccc |
| Ndufb9 | Forword primer:agaatcagcatcctcagccg Reverse primer:cttaacctcccgatcccagc |
| Suclg1 | Forword primer:gccaagcctgtagtgtcctt Reverse primer:atggtagttcccagctgtgc |
| Glrx5 | Forword primer:aggacaaggtggtggtgttc Reverse primer:ctcgccgttgaggtacactt |
| Cox6b1 | Forword primer:tccccaaccagaaccagact Reverse primer:ttcagctatgcggtcatccc |
| TCF7L2 | Forword primer:tggagggctctttaagggg Reverse primer:gatccgttggggaggtagg |
| FOXJ3 | Forword primer:tcactgtctcacccccagat Reverse primer:ctgatggttcggatggtgct |
| TCF7 | Forword primer:ttgatgctaggttctggtgtacc Reverse primer:ccttggactctgcttgtgtc |
| HINT1 | Forword primer:ggtgccttgctttccatgac Reverse primer:attcaggcccagatcagcag |
| TOMM7 | Forword primer:aagctgagcaaagaggccaa Reverse primer:ccccaaagtaggctcaaaac |
| GUK1 | Forword primer:ttcagcgtgtcccataccac Reverse primer:gcacacagatgcggttcatg |
| SLC25A5 | Forword primer:gcttccggatcccaagaaca Reverse primer:caagcgtgcctgtgtacatg |
| NUDFB1 | Forword primer:tacttcagattgtgcgggacc Reverse primer:cttcactgggttgcaattccc |
| ATP5MG | Forword primer:acccctgctgagatccctag Reverse primer:gccccgcttgcctataatct |
| COX7A2 | Forword primer:ttgggcagaggacgataagc Reverse primer:gaagccacagccagctcata |
| MPC2 | Forword primer:taccaccggctcctcgataa Reverse primer:gtttttctgcaggtctggcc |
| PMPCB | Forword primer:gctggaggtgtttcccatga Reverse primer:gcccaaccaacagcttcaac |
| FAM210B | Forword primer:gtgtggacatgcctgcaatc Reverse primer:agggcacagagactagcgta |
| NDUFC1 | Forword primer:ccttgctgcgtccccttt Reverse primer:acaagaagacagtggtgccc |
| NDUFB9 | Forword primer:cgtcagcatccacagccata Reverse primer:cctccgcagtttcttccact |
| SUCLG1 | Forword primer:ttcctacacagcttctcggc Reverse primer:cctttccctggagtggttcc |
| GLRX5 | Forword primer:gtggtggtcttcctcaaggg Reverse primer:tgaggtacacttgcgggatg |
| COX6B1 | Forword primer:tccccaaccagaaccagact Reverse primer:cgttgctcatcccagtctgt |
